# A Glimpse into Gynecologic Practice During the Islamic Golden Age

**DOI:** 10.1007/s43032-023-01423-5

**Published:** 2024-01-02

**Authors:** Hossam E. Fadel, Ayman Al-Hendy

**Affiliations:** 1 Maternal Fetal Medicine, Department of Obstetrics and Gynecology, The Medical College of Georgia, Augusta University, Augusta, GA USA; 2 Department of Obstetrics and Gynecology, University of Chicago, Chicago, IL USA

**Keywords:** Islamic civilization, Medieval medicine, Gynecologic practice, Medical history

## Abstract

The Islamic Golden Age was the time in history from eighth to fourteenth century. This era was marked by expansion of Islamic world to all the Middle East, North Africa, South and East Europe, and Central Asia. The Islamic world was the wealthiest region in the world at that time and that wealth was utilized to promote great flourishing in the arts, philosophy, science, and medicine. The practice of healing was considered the most noble of human undertakings by Islamic scholars. In this era, many great physician-scientists emerged in the Islamic world, albeit several were not Muslims, who examined prior writings, corrected many, and proceeded to produce their own observations and innovations. This article highlights some of the most important contributions to gynecology of some prominent scholars during this shining phase of medical history.

## Introduction

Islamic teachings inspired Muslims to acquire knowledge from all sources available at that time primarily from Greek, Roman, and Persian philosophers, and scientists such as Hippocrates, Aristotle, and Galen. Their books were translated from the Greek, Syriac, Persian, Egyptian, Sanskrit, into Arabic [1]. The Muslims added a lot of their own observations, experiences, experiments, and innovations [2]. They made a lot of corrections to the older ideas and came up with multiple new theories and branches of science particularly in astronomy, mathematics, and medicine [3, 4]. The associated Muslim civilization that supported such knowledge surge, commonly referred to as the Islamic Golden Age, spanned almost 700 years beginning in the seventh century C.E. [5].

Many physicians of this era wrote books incorporating their own observations based on their clinical practice as well as experimentation. These prominent physician-scientists include but not limited to as follows: al-Razi (Rhazes, 841–926 CE), al-Zahrawi (Albucasis, 930–1013 CE), al-Majusi (Haly Abbas, died 994 CE), ibn Sina (Avicenna, 980–1037 CE), al-Baladi (early eleventh CE), ibn Maimon (Maimonides, 1135–1204 CE), al-Dhahabi (died c 1348), Areeb al- Qurtuby (912–970 CE), and al-Akhawayni (c 938). Their books were translated into several languages, including Latin, and were used for teaching in European medical schools up to the eighteenth century [6–9]. A short biography of these scholars has been reported [3] and in a previous article [10].

In this article, we will highlight a sample of gynecological concepts and practices in the Islamic Golden Age that were state of the art in the world at that time and in many instances were accurate and paved the way to some contemporary notions and dogma. In some instances, in parentheses, we added what we think is the modern meaning and/or significance of these scholars’ statements.

## General Medical Practice Principles

Many female physicians existed in the Islamic Golden Age era [8], who treated members of both sexes [11]. In case of disease, men can treat women, and women men, even if this means that they must expose their genitals to a stranger of the opposite sex. This is strictly prohibited in any other situation. This permissibility is based on the principle of *darūra* (necessity), an Islamic jurisprudence rule which indicate that “necessities override prohibitions” [12].

Ibn Sina’s (Avicenna) advice, in his book *al-Qanun Fi al-Tibb* (*The Cannon*), on the management of the terminally ill patient and end of life management is amazingly aligned with contemporary practices: “Up to the last moment we should endeavor to soothe, but we must not gamble with a life by powerful remedies or big operations where there is no well-founded hope” [13]. Al-Zahrawi and ibn Sina appreciated the anti-septic properties of alcohol and used them extensively in the dressing of wounds (more than 800 years before Pasteur started talking about germs) [14].

Ibn Sina advocated the use of “anesthesia” for painful applications and procedures. He had several recipes for effective oral anesthetics. He said: If it is desirable to get a person unconscious quickly, add sweet smelling moss to the wine. If it is desirable to procure a deeply unconscious state, place darnel-water into the wine; or administer fumitory, opium, hyoscyamus (half-dram dose of each); nutmeg, crude aloes-wood (4 grains of each) and add this to the wine. Another method he used is to boil black hyoscyamus in water, with mandragora bark, until it becomes red and then add it to the wine. It is noteworthy that he relied on wine, consumption of which is prohibited in Islam. But because it was the best available choice at the time, it was acceptable to use, based on the above-mentioned Islamic jurisprudence rule “necessities override prohibitions” [12].

## Uterine Tumors

Al-Razi in his book, *al-Hawi fi al-Tibb* (*The Continens*), described uterine tumors and called them “waram liefy.” In Arabic, this means a tumor full of “*Lief*,” i.e., fibers (most likely what we now call fibroids or leiomyoma) [15]. He described their pressure symptoms: frequent urination, difficulty emptying the bladder, and constipation. He also explained the different shapes of the uterus in the presence of fibroids depending on their position. He also was able to identify the cervical location of such tumors as a painless lesion—attributing this to its low sensitivity unless it causes pressure symptoms with difficulty in evacuation either of the bladder (urination) or the rectum (defecation). Also, he described the neurological pressure symptoms of lateral large fibroids demonstrated by shooting pain through the legs (maybe what is now called sciatica due to pressure on the sciatic nerve).

## Gynecologic Cancers

Al-Razi described ulceration of the neck of uterus secreting pus (which we now recognize as cervical cancer). He also described its local infiltration of pelvic and abdominal structures. He also stated that such a malignant tumor led to a fixed non-mobile uterus [15].

## Reproductive Physiology, Menstruation, and Its Disorders

Al-Razi differentiated, via thorough history taking, between the bleeding hemorrhoids versus menses, noting the periodicity of the menses bleeding versus the absence of pain with hemorrhoids [15]. Ibn Sina (Avicenna) recognized that the uterus moves/contracts during intercourse (likely what we now recognize as orgasm). He also indicated that after ejaculation, the neck of the uterus (the cervix) moves further down to suck the semen towards it [13]. Al-Zahrawi in his book *al-Tasrif* said that the best age of pregnancy is from 15 to 40. He also had a detailed description of the uterus and its neck (cervix). He described that the uterus ends up with two corners or horns (which were later called Fallopian tubes) that end up in the ovaries. He also stated that the ovaries push their “fluid” into the inside of the uterus where it meets the “fluid or semen” of the man and that how pregnancy starts [16]. Al-Zahrawi was the first to indicate that the pregnant uterus contracts and relaxes. He said specifically that it relaxes to allow the pregnancy to grow (effect of progesterone) and contract during labor (effect of oxytocin and prostaglandins) to push the baby out [16]. Al-Zahrawi also nicely classified amenorrhea into normal or abnormal. Amenorrhea is normal if the girl is not mature yet. Menses usually start around 14 years but can be a little before that or a little after (puberty). Amenorrhea is also normal during pregnancy and lactation, and as a woman gets old (menopause). He suggested the age of menopause to be around 45 years but said it can vary: a little before or a little after. He described abnormal amenorrhea as either menses that is infrequent and slight in amount (oligomenorrhea) or completely absent. He attributed abnormal amenorrhea to “something wrong” with either the structure of the uterus or its blood vessels [16]. Ibn Sina also commented about malnutrition-related amenorrhea and that very thin women do not menstruate (which is equivalent to what we recognize now in anorexia nervosa and related eating disorders or what is occasionally seen in athletes) [13]. Musa ibn Maimon (Maimonides) mentioned that amenorrhea can result in serious consequences such as feeling pins and needles in the limbs, backache, visual disturbances, or swelling of the breasts and appearance of milk secretion (which is now recognized as galactorrhea, and it is present in amenorrhea associated with high serum prolactin levels typically due to pituitary gland adenoma) [17]. He stated that if the menstrual flow is very heavy, it will lead to paleness of the face (anemia), swelling of the legs, and bloating of the entire body (likely referring to generalized edema secondary to severe hypoproteinemia).

Physicians in the Islamic Golden Age described a condition called *ikhtināq al-rahim* (or uterine suffocation) when the uterus is full of menstrual blood that causes severe spasm and severe pain and can disturb certain mental functions (and hence the word hysteria, hyst = uterus in Greek) that is still in use today. Physicians thought that lack of intercourse was one of the possible causes of this condition as the uterus is in want of semen and keeps wandering in the woman’s body. Therefore, young, unmarried women, and widows who do not engage in sexual intercourse were particularly prone to this disease [11].

A special type of amenorrhea, hypomenorrhea, and oligomenorrhea has been recognized by the scholars of the early Islamic period [13, 16–18]. Ibn Sina described women that look and behave like men, and they are obese and muscular (most likely referring to women suffering from what we now call severe polycystic ovary syndrome, PCOS [18]). Musa ibn Maimon added a more specific description of these women as follows: there are women whose skin is hard, and whose nature resembles the nature of a man, their voice is loud and they are generally big, they can become bearded and may not menstruate at all [17]. Al-Zahrawi suggested that amenorrheic obese women can get better with weight loss, fasting, eating less, eating good food, and exercise (not different from current modern approach to PCOS patients) [16]. Many scholars in the Islamic Golden Age described various herbal combinations to help these women patients with PCOS to reduce their weight resulting in return of regular menses and consequently pregnancy.

## Puberty and Its Disorders

Ibn Sina described the condition of *Ritqa* (Imperforate Hymen): there is something on the mouth of the genitals whether it a muscle or a strong membrane that prevents intercourse, prevents the flow of menses, and prevents pregnancy. It is usually there from the beginning but becomes visible when the woman starts her menarche. Because of this condition, menses cannot find an exit, and menstrual blood accumulates in the vagina and then backs into the uterus, so the woman will have severe pain and major disease. The woman turns black and then suffocate and perish (He might be referring to septic shock syndrome secondary to hematometra, secondary infection, and subsequent pyometra. The death may also be caused by the development of uremia secondary to back pressure of the hematocolpos on the ureters or the associated urinary retention) [13]. Al-Razi accurately described how to diagnose imperforate hymen and indicated that its treatment should take place before the patient reaches puberty as it is very painful and sometime could be fatal if left untreated. Ibn Sina described the treatment as follows: if the *Ritqa* is visible, it should be cut in the middle of genitalia until the two sides are separate. If there are extra tissues, they should be removed but not taken from the original genitals. Then one should leave a piece of wool soaked with oil and alcohol for 3 days and ointment should be added regularly to avoid refusion or narrowing [13]. This is a very insightful step in the treatment which is applicable to all procedures where an opening is made, or to correct obstruction.

## Contraception

In ibn Sina’s opinion, controlling one’s fertility is a justifiable application of medicine. He mentioned that a physician may consider the prevention of pregnancy in a young woman, if pregnancy is feared due to an ailment in her womb, or if the woman had encountered damage of her bladder after a previous delivery. A subsequent delivery may make this damage permanent, causing leakage of urine and failure to maintain continence until the end of her life. Ibn Sina is credited for associating obstructed labor with the large size of the fetus and to be the first to associate difficult labor with urinary fistula formation and subsequent urinary incontinence [13, 19]. Ibn Sina said that to achieve contraception, methods with high failures should be avoided. To prevent pregnancy, he recommended that “The two emissions should not occur together, and the male should withdraw quickly” (the two emissions are the male ejaculation and the orgasm in the female, as he believed that it causes her to push the ovarian fluid into the uterus to meet the male semen there). He also stated: “after completion (of the intercourse), the woman is asked to stand upright and to hop backwards seven or nine times as this might expel the semen. Also sneezing might help expel the male semen.” Other measures he recommended is that the woman might consider to insert (vaginally) tar before and after coitus. Other recommendations were to anoint the penis with tar, balsam, and ceruse lead, or to insert, before and after coitus, oil of pomegranate and alum. He stated that “The insertion of a cabbage bud and cabbage seeds before and after coitus is an effective contraceptive especially if these are dipped in tar or menthol oil before insertion in the vagina. An alternative option is to insert leaves of willow mixed in equal parts of oil of colocynth, white jalap, sulfur, scammony, cabbage seed and tar.” Other recipes were the insertion of pepper after coitus, or the insertion of elephant’s dung. Ibn Sina also prescribed the insertion of ivy leaves in the vagina outside of menses. Ibn Sina also recognized that taking certain drinks orally can prevent pregnancy (probably first mention of an oral method of contraception as all other methods used up to that point were methods that are applied locally to or around the genital organs). As an example, he recommended drinking three ounces of basil [13].

Abubakr al-Akhawayni known as “Abu Hakim” (Father of Wisdom) of the tenth century, a student of a student of al-Razi, wrote a book called *Learner Guide to Medicine* [20]. In the book, he described a barrier method for contraception by placing a barrier made of “Mazu” (Oak tree sap) at the far end of the vagina (in front of the cervix) to prevent semen from entering the uterine cavity. He explains that this device should be connected to a thread for easy removal after intercourse (likely the first prototype of a removable contraceptive device as a vaginal diaphragm).

Physician-scientists in the Islamic Golden Age may have developed the world’s first condoms [21]. It is unclear where the word condom came from, but one likely origin is the Persian word *Kondu* meaning a long storage vessel made of animal gut. The word then came through Latin into English as condom. A penile sheath made of animal gallbladder was first advocated by al-Akhawayni and likely represents the first prototype of the modern condom.

Al-Akhawayni also recommended that men remove the penis rapidly from the vagina at the peak of excitement so as to ejaculate outside the vagina. This was practiced prior to Islam and continued afterwards since Prophet Mohammad (PBUH) did not prohibit it [22] (this method is still a recognized method of contraception: withdrawal method or coitus interruptus). Al-Akhawayni also recommended the application of some topical vaginal agents (before or just after intercourse) which he believed to have spermicidal characteristics.

## Male and Female Infertility

Several scholars during the Islamic Golden Age recognized that infertility can be caused by male infertility, female infertility, or a combination [23, 24]. Scholars of that period described the importance of good *Mizaj* (temperament) to produce good quality semen. They believed that fear, anger, or sadness will disrupt the *Mizaj* and can lead to male infertility. To diagnose male infertility, they placed male semen gently on surface of water; if it floats, it is likely associated with infertility. It was understood that ovaries in the female are the equivalent to testicles in the male and that the ovaries produce the female fluid which is transported to the cavity of the uterus via the horns of the uterus where it mixes with male semen. They recognized that hard swellings of the uterus (most likely uterine fibroids) can cause infertility by preventing the mixing of male and female semen. Disrupted *Mizaj* of the uterus can cause infertility as it has been associated with less frequent menses (oligomenorrhea, probably a reflection of patients having PCOS among other reasons), heavy menstrual bleeding (probably associated with uterine fibroids), or hypomenorrhea or amenorrhea (probably in patients with hypothalamic disorders or premature ovarian failure). Another cause of female infertility was presumed to be obstruction in the female genital tract preventing the normal meeting of the male and female fluids. Therefore, they devised testing for this cause. A garlic wrapped in a piece of cloth was placed in the woman’s vagina at night before sleep. If she smelled it or tasted it in her mouth the next morning, it was a sign of lack of obstruction in the woman’s genital tract and that semen also can pass. Another version of the same concept was to have the female sit on a seat with a hole in its middle, put some perfumed materials (*bokoor*) underneath and surround the patient with air-tight tent-like clothing from the neck down. If she can recognize the smell of the specific perfume or scent that suggests that there is no obstruction in her genital tract.

An association between obesity and female infertility was first described by physicians in Islamic Golden Age including al-Razi, al-Zahrawi, ibn Sina, and al-Akhawayni [20, 23–25].

Al-Razi, in his book, *al-Hawi* (page113), states that “if the uterus is affected by cold *Mizaj* (disease) then that closes the vessels, and the woman will become infertile, and these women have no menses (amenorrhea) or little menses (hypomenorrhea/oligomenorrhea)” [15]. In page 104, he said that “the amount of both semen and menses are important for fertility potential and whenever either or both are low or minimal, that can cause infertility.” In page 86, al-Razi states that “women who do not have menses are usually unable to get pregnant.” In page 93, he said that “infertility also can happen when there is infrequent intercourse so the woman fluid which have the female seed will dry out.” He also says that “lack of exercise and fatigue can cause infertility as the secretion at the uterine neck (cervix) become less attracting to the man semen and as such the semen does not stick to the uterine cervix and fall out.” Al-Razi also connects obesity to infertility (probably one of the first to do so in history); “Obese women are unlikely to get pregnant and if they do, they miscarry, or she will have a difficult labor.” He treated that by helping the woman lose weight. Al-Razi also said that “female infertility can result from blockage of the mouth of the uterus (cervix) by thick secretions” (thick cervical mucus is recognized as a cause of infertility). In this situation, he recommended that the patient should drink lots of fluids with herbs like cumin and karafs, as well as eat cabbage then bathe in warm water. Al-Razi also said: women’s best fertile years are between 15 and 40 years of age. “If a girl gets pregnant earlier, she may perish as her uterus (probably meaning her pelvis) is too small.” Al-Razi is also probably the first to connect depression and psychological status of a woman to her fertility potential. On page 108, he says “The woman who is always sad and miserable will not get pregnant.” Al-Razi stated that “women do not get pregnant if they have intercourse at the beginning of menses as the uterus is full of blood and is humid.” (The observation is accurate but the real cause as we know it now is that the ovum is not released from the ovary at that early stage of the menstrual cycle) [15].

The third chapter of ibn Sina’s book *al- Qanun fil-Tibb* beginning from Fan 21 is totally devoted to infertility [13]. Ibn Sina says that infertility is caused by either something wrong in the man’s semen or the woman’s semen, or their organs (uterus or penis), or by general external reasons such as depression or obesity. He also stressed the importance of compatibility and that some couples cannot get pregnant as their semen are incompatible, and that they can go on and marry others and have no problem having children with their new spouses (which is likely the first recognition in history of what is now accepted as immunologically mediated infertility and recurrent pregnancy loss) [13]. Ibn Sina stated that infertility can also happen if there is a disorder in the uterus such as a tumor, ulcer, or a barrier at its mouth (cervix) that prevents the passage of man’s semen inside the uterus. He also mentions that infertility can happen if the man and woman reach orgasm (*ishtemal*) at different times. He explained that if the man ejaculates first, then he will leave the woman and she will not reach orgasm. If the woman reaches orgasm first, then when the man eventually ejaculates, there will not be that movements of the uterine mouth (cervix) that normally function to attract and suck the man’s semen inside the uterus. Ibn Sina stressed the importance of foreplay in allowing the man and woman to synch their orgasms. “The husband should touch the wife breasts gently, and also he should touch her pubic region especially between the two labia as this is her satisfaction spot” (which is now recognized as the clitoris and indeed likely the most sexually sensitive spot in the female body). Ibn Sina continues, “then he should start the intercourse and when her eyes get congested, her breathing become faster, and her talk become so wet then he should ejaculate as close to the uterine cervix as possible. The man should not withdraw right away and after he withdraws, the woman should stay flat on her back and close her thighs and better if she falls asleep afterwards”.

Ibn Sina rejected numerous prior fertility tests such as floating the man or woman ejaculates on water and whichever float and does not sink, the infertility is from his or her side, or that each spouse should urinate on two different specific seeds and watch them for 7 days and if the seeds start to germinate, then that specific spouse is fertile [13]. However, ibn Sina accepted the “garlic or fume test” described above. If she fails the test, then there is blockage or bad fluids in her uterus.

Abbas al-Majusi devoted the 24th chapter, article 8 of his book to infertility. He divided the causes of female infertility in three categories: blockage or obstruction of the mouth of the uterus, bad *Mizaj* (disease) of the uterus or incompatibility of the man and woman semen [26]. Then he elaborately described detailed herbal recipes for each scenario which can be used orally or mixed in a piece of cloth and placed inside the vagina to reach the uterus or as a vapor (bokhoor).

Abu Al-Qasim al-Zahrawi (Abulcasis) in his book *al-Tasrif* differentiated between irreversible infertility (*al-O’qm*) versus delay in getting pregnant [16]. He stated that causes of female infertility are either a disease in the uterus being too dry or too humid, absence of menses (amenorrhea), a blockage in the uterus, retention of the menses blood, very thick cervical secretion, obesity, a tumor in the uterus, or an ulcer(on its neck). He described external causes of infertility such as women refusing sexual intercourse or standing up very soon after orgasm.

Like others, al-Zahrawi used the garlic or fume test mentioned above to test for genital tract patency and hence fertility. It is remarkable that al-Zahrawi in Andalusia, al-Razi in Syria, and Ibn Sina in Persia all described fairly similar versions of this “garlic or fume test” of genital tract patency suggesting that cutting edge physician-scientists in the vast Muslim world tried to stay updated of latest developments in their field not unlike current times.

Al-Zahrawi described some fertility enhancing medications, for example, the woman drinking or eating elephant ivory mixed with honey. Another al-Zahrawi recommendation was for the woman to put a pigeon gall bladder in a piece of wool and put it in her vagina after she finishes menses for 3 hours then take it out and have intercourse. He also cautioned that sometimes infertility cannot be corrected like a tree that does not bear any fruits [16].

## Surgical Innovations and Development of Instruments

**Fig. 1:**
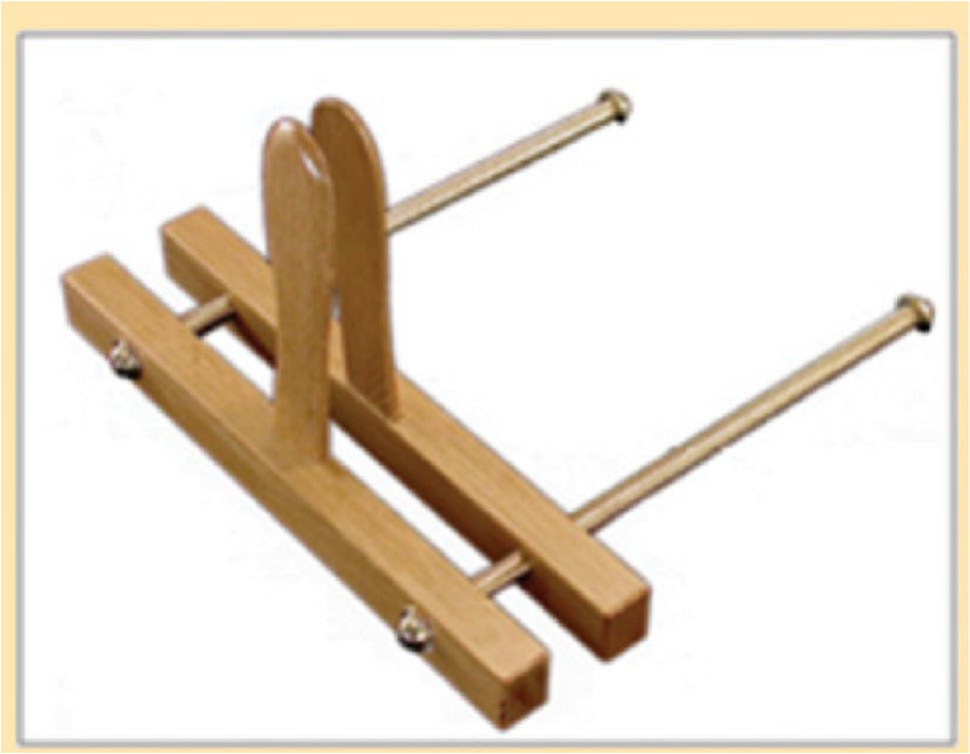
Vaginal speculum (al-Zahrawi design), original undated instrument in Frankfurt Museum for the History of Science and Technology in Islam. Photo courtesy of Dr. Fuat Sezgin, the director of the museum. Source: MN Saad, *op. cit*

Al-Zahrawi managed for the first time in history to present surgery in a very knowledgeable fashion and is referred to as the father of modern surgery. He demanded a lot from individuals who wanted to be surgeons. He stated: “This is the reason why there is few skillful operators in our day; the art of surgery is long and it is necessary for its exponent before he exercises it to be trained in anatomy, so that he may be fully acquainted with uses, forms and temperament of the tissues, how they are joined and how they may be separated, that he should understand fully also the bones, tendons and muscles, their numbers and their attachment and also the blood vessels, both arteries and veins with their relations” [16]. As a result, surgery, which up till then had been left to cuppers and barbers, was, thanks to al-Zahrawi, completely integrated into scientific medicine. His book *Kitab al-Tasrif* which was translated to Latin had a great influence on the subsequent surgical work and teachings well into the eighteenth century. Surgical suturing was mostly developed in the Islamic age. Al-Zahrawi was the first surgeon to use animal gut to suture a wounded intestine. He understood that a compatible biomaterial should be used inside the human body so that the body would not reject it. He recognized that animal gut is a dissolvable material after he discovered that a monkey had eaten the strings of his lute—he checked the monkey’s excreta for several subsequent days and did not find the lute strings. Suture strings are still made of animal gut even today. Other suturing materials included flax, horsehair, silk, and wool. He was also the first to use cotton to stop bleeding and linen for dressing. Al-Zahrawi was the first to describe and use a vaginal speculum (Fig. 1) for gynecologic examinations and procedures [16, 27]. He illustrated the speculum in his book (*Al-Tasrif*). [16]. As a matter of fact, he was the first to include drawings of surgical instruments in books. His book had pictures of about 200 instruments for different branches of surgery, general surgery, dentistry, ophthalmology, orthopedics, gynecology, and obstetrics. Most of these instruments were invented by him [16]. Other physicians in the early Islamic era used the vaginal speculum and it was called by Arabic names:* Lawlab* (screw), also *Miftah al-farj* (the key to the vulva or vulvar opener). Ali ibn Abbas al- Majusi described how to use the speculum in situations related to gynecological diseases and childbirth [26]. In his book, Ibn Sina for the first time described the use of a mirror to reflect the light with the vaginal speculum to better examine the vagina and cervix and to also treat diseases in that area such as abscess and tumors [13]. He also described that the size of the speculum to be used should be based on the patient’s age and body type. Serefeddin Sabuncuoglu (1385–1470) was a prominent follower of al-Zahrawi. In his book *Imperial Surgery*, he went beyond drawings of the surgical instruments to actual drawings of procedures and how the instruments were used. The position of the patient was also described in detail in text and drawings. For example, he described that the patient would sit in a U-shaped seat, flex her knees, and hold her legs and thighs against her abdomen (lithotomy position) for proper examination of the vagina and cervix and for procedures on these organs [28].

## Pelvic Organ Prolapse

Maimonides described probably for the first time that uterine prolapse occurs in women who were pregnant many times especially if their deliveries have been difficult. This is brought about by the ensuing force of expulsion which weakens the pelvic muscles [17].

## Conclusion

This article was meant to address a major gap in knowledge. There were numerous significant contributions to the field of medicine by the physician-scientists belonging to various religions but all working within the Islamic world during the glorious era called Islamic Golden Age. In this manuscript, we wanted to highlight particularly their contributions to the specialty of gynecology to complement our previous sister article where we focused on obstetrics [10]. Unfortunately, historians generally have ignored such contributions of Muslim scholars to the Renaissance. They called the period between Ancient Greek civilization and the Renaissance “The Dark Ages,” which indeed it was in Central and Western Europe (aside from Andalusia) but parallelly there was a great civilization that existed right to the east and south during the Islamic Golden Age. Fortuitously, objective historians recently started to unearth and report the great scientific contributions of Islamic civilization [29–31] particularly in medicine. In this article, we wanted to briefly highlight the status of gynecologic practice in this era and the seemingly good and occasionally impressive understanding of some physiological facts and pathological conditions of the female genital tract.

## Data Availability

Not applicable.
